# Thrombopoietin: tickling the HSC's fancy

**DOI:** 10.15252/emmm.201708450

**Published:** 2017-11-30

**Authors:** Ah Ram Kim, Vijay G Sankaran

**Affiliations:** ^1^ Division of Hematology/Oncology Boston Children's Hospital Harvard Medical School Boston MA USA; ^2^ Department of Pediatric Oncology Dana‐Farber Cancer Institute Harvard Medical School Boston MA USA; ^3^ Broad Institute of MIT and Harvard Cambridge MA USA

**Keywords:** Genetics, Gene Therapy & Genetic Disease, Vascular Biology & Angiogenesis

## Abstract

Thrombopoietin (THPO) has been well characterized as a key regulator of platelet production. THPO also plays an important role in the maintenance and regulation of hematopoietic stem cells (HSCs). In this issue of *EMBO Molecular Medicine*, Pecci *et al* (2018) describe a newly identified homozygous mutation in *THPO* causing congenital amegakaryocytic thrombocytopenia, a disease characterized by a significant impairment in platelet production with rapid onset of aplastic anemia within a few years. The paper nicely investigates the underlying pathogenic mechanisms of this disease. Importantly, this study, in tandem with other recent ones, shows that this rare genetic form of aplastic anemia is treatable with THPO receptor agonists, emphasizing the paramount role of genetic testing in cases of aplastic anemia and other bone marrow failure disorders. This report also refines our understanding of the role of THPO in human HSC function and illustrates the important biological insight that can be gained through studies of such rare genetic disorders.

While model organisms have provided considerable insight into physiologic processes, such as hematopoiesis, it is clear that there are many limitations in our ability to extrapolate these findings to human biology. A classic example of this is the variable requirement for the key hematopoietic cytokine, thrombopoietin (THPO), in mice as compared with humans. Thrombopoietin was initially identified as a key regulator of platelet production from precursor megakaryocytes (Kaushansky, 2006). Thrombopoietin is also able to promote the formation of megakaryocytes from earlier hematopoietic progenitor cells. Thrombopoietin acts in a manner similar to other hematopoietic cytokines, including erythropoietin and granulocyte colony‐stimulating factor, by binding to and promoting homodimerization of its receptor, MPL, which then activates downstream signaling pathways through the receptor‐associated tyrosine kinase, JAK2 (Fig 1; Kaushansky, 2006). Importantly, THPO has been shown to have key and non‐redundant roles in the maintenance of hematopoietic stem cells (HSCs) and other early progenitors. Interestingly, while THPO can stimulate human and mouse HSC expansion, knockout mice for either *Thpo* or *Mpl* show an approximately sevenfold decrease in transplantable HSCs, but do maintain relatively normal cellularity in the bone marrow and with the exception of the thrombocytopenia, there are no other peripheral blood abnormalities present (Solar *et al*, 1998). In contrast, humans with total loss‐of‐function of *MPL* have congenital amegakaryocytic thrombocytopenia (CAMT) that involves severe thrombocytopenia due to impaired megakaryocyte production evolving to trilineage marrow aplasia within the first years of life (Ihara *et al*, 1999; Ballmaier & Germeshausen, 2009). Such phenotypic differences between mice and humans emphasize the value of studying human variation in hematopoiesis.

**Figure 1:**
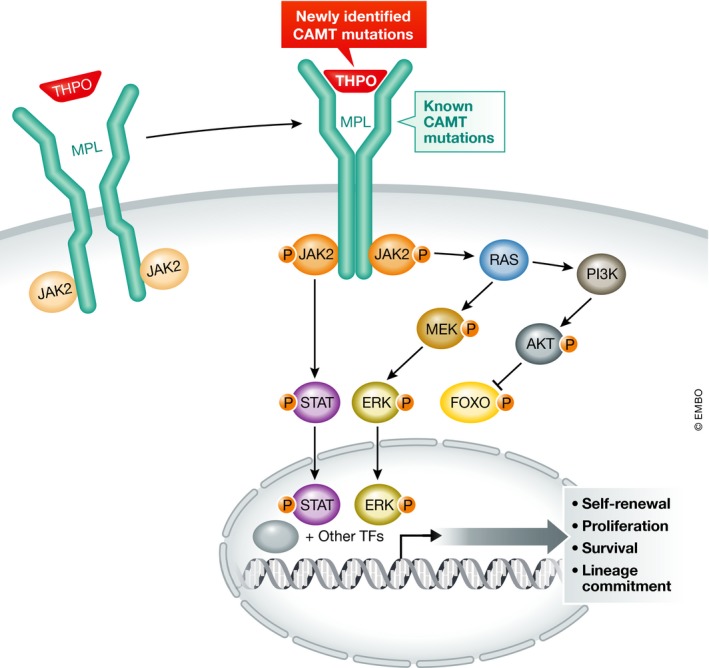
Mechanisms of thrombopoietin signaling in HSCs and other progenitors THPO binds to its receptor, MPL, resulting in homodimerization of MPL. Dimerization of MPL leads to activation of multiple downstream signaling pathways as shown, predominantly through the receptor‐associated tyrosine kinase, JAK2. Activation of these pathways supports the self‐renewal, proliferation, survival, and differentiation of HSCs and also supports megakaryocyte/platelet production. Previously, mutations in *MPL* have been reported as the predominant cause of CAMT. Recent studies of CAMT cases with *THPO* mutations have been reported here and elsewhere. These cases are distinct from other causes of CAMT in that they can be treated with MPL agonists.

The majority of CAMT cases are due to mutations in *MPL*. Until recently, no other mutations have been identified that cause this condition. However, three recent papers have now identified a new mechanism by which CAMT can arise, and a distinct treatment approach for these cases. Homozygous mutations in *THPO* can result in CAMT that evolves rapidly to aplastic anemia (Dasouki *et al*, 2013; Pecci *et al*, 2018; Seo *et al*, 2017). In this issue, Pecci *et al* (2018) identify a homozygous mutation in *THPO* (chr3:184091244 C>T in hg19 that results in a missense change of p.R119C) in a family where multiple children have CAMT. The mechanistic follow‐up studies performed have helped to extend our understanding of how CAMT can arise in these cases. Moreover, the findings have resulted in effective and targeted therapeutic intervention.

Using functional follow‐up studies, the authors show that the mutations impair secretion of THPO and this is the major cause of the CAMT. The authors show that the identified mutations do not appear to cause instability or decreased production of THPO, but rather that there is a still undefined aspect of secretion that is impaired by the missense mutations identified in these cases. In these patients, the serum levels of THPO are extremely low, which supports this finding. Importantly, given the identification of these mutations and the characterization performed, the authors in this case were able to ameliorate the pancytopenia present through treatment with the MPL agonist, romiplostim. These results extend the recent successful treatment of two other patients with CAMT due to homozygous *THPO* mutations with romiplostim and definitively define this condition as a treatable form of aplastic anemia (Seo *et al*, 2017). In the future, it will be critical for any patients with aplastic anemia and preceding thrombocytopenia to be screened for *THPO* mutations, as this condition can be readily reversed with clinically available MPL agonists.

The cases described in this paper and in the previous studies all have reduced serum levels of THPO, whereas ordinarily THPO levels should be elevated in cases of aplastic anemia or other forms of thrombocytopenia (Dasouki *et al*, 2013; Pecci *et al*, 2018; Seo *et al*, 2017). This finding contrasts with a recent report of mutations in the cytokine erythropoietin that allow for effective synthesis and where serum levels were elevated in the patients, but downstream signaling was impaired as a result of altered binding properties by the mutated ligand (Kim *et al*, 2017). It is likely that additional *THPO* mutations will be identified in the future; it will be interesting to examine whether any of the mutations that are identified in future studies will qualitatively alter the cytokine, rather than impairing secretion. Such mutations would help in better defining the signaling mechanisms by which THPO can stimulate HSC function and megakaryocyte/platelet production. These studies and other recent papers also emphasize the spectrum of phenotypes that can arise from perturbations in this pathway. For example, monoallelic loss‐of‐function of *THPO* can cause isolated thrombocytopenia (Noris *et al*, 2017) and it is likely that further genetic studies may define a broader series of alleles in this pathway leading to a range of diverse phenotypes.

As further population‐based studies are performed that provide insight into normal and pathogenic variation in human hematopoiesis, important new lessons will likely emerge as to how the THPO pathway can best be modulated (Ulirsch *et al*, 2016; Guo *et al*, 2017). Importantly, this new insight will not only provide a paradigm for understanding how this pathway can be perturbed in rare conditions, such as CAMT, but will also provide insights into approaches to modulate this signaling pathway for treating more common conditions, such as idiopathic aplastic anemia, where MPL agonists are already showing efficacy (Townsley *et al*, 2017). The work from Pecci *et al* (2018) in this issue and the other recent related articles provide the first steps in this direction and more generally demonstrate how naturally occurring variations can enable us to gain important insights into human biology.
